# Nonmetallic Atom Regulation for Enhanced NiPr Monolayer Sensing of SF_6_ Decomposition Gases: A DFT Study

**DOI:** 10.1021/acsomega.6c01664

**Published:** 2026-05-05

**Authors:** Xianhao Long, Xing He, Hongxing He, Yi Zhang, Mingyang Xiong, Enrui Dai, Zhifeng Nie

**Affiliations:** Yunnan Key Laboratory of Metal−Organic Molecular Materials and Device, School of Chemistry and Chemical Engineering, 162634 Kunming University, Kunming 650214, China

## Abstract

The identification and mitigation of SF_6_ decomposition gases (SO_2_, H_2_S, HF, SOF_2_, and SO_2_F_2_) are critical for environmental protection and human health. In this study, we utilized density functional theory (DFT) to systematically investigate the adsorption characteristics and sensing properties of these gases on pristine nickel-porphyrin (NiPr) and nonmetal-atom-modified nickel-porphyrin (NiNPr, N = B/O) monolayers. The studied results indicate that the NiPr monolayer is not suitable as a gas-sensitive material for the detection of SF_6_ decomposition gas molecules due to its weak adsorption ability and low sensitivity. However, the incorporation of nonmetallic B and O atoms into the NiPr monolayer significantly enhances the binding energy, ensuring the structural stability of the resulting NiBPr and NiOPr monolayers. The bandgap values of these doped monolayers are reduced to 0.326 eV (NiBPr) and 0.057 eV (NiOPr) compared to 0.985 eV for the pristine NiPr monolayer. Notably, the NiBPr and NiOPr monolayers demonstrate enhanced adsorption energy for SO_2_, H_2_S, SOF_2_, and HF gases (−0.56 eV to −1.08 eV), indicating chemical adsorption. A comprehensive analysis of the total electron density distribution, charge density difference, and density of states was conducted to elucidate the interaction mechanisms between the gases and the monolayers. Additionally, significant changes in work function and bandgap values confirm the high sensitivity of these monolayers. Recovery time assessments for gases including SO_2_, H_2_S, HF, SOF_2_, and SO_2_F_2_ reveal that the NiBPr monolayer is viable as a reusable gas sensor for SO_2_, H_2_S, HF, and SOF_2_, while the NiOPr monolayer is apt for sensing H_2_S, HF, and SOF_2_ gases. These findings are significant in highlighting the potential of NiBPr and NiOPr monolayers as innovative sensors for the SF_6_ decomposition gases.

## Graphical abstract

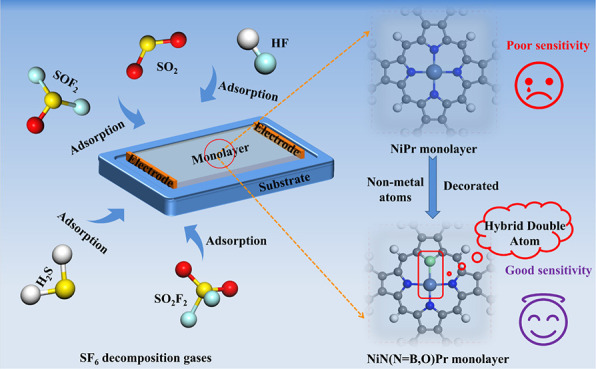

## 1 Introduction

Sulfur hexafluoride (SF_6_) is widely used in power system insulation equipment because it has acute arc extinguishing ability and insulation performance.^1–4^ It is known that although SF_6_ is chemically stable at room temperature, partial discharge or overheating will inevitably occur in some equipment during practical applications,^5–7^ which will lead to the decomposition of SF_6_ into different gases such as SO_2_F_2_, H_2_S, HF, SO_2_, and SOF_2_.^8,9^ If SO_2_F_2_, H_2_S, HF, SO_2_, and SOF_2_ gases are not handled properly, they will not only inflict harm on the environment but also pose a threat to human health.^10–13^ Therefore, monitoring or elimination of SF_6_ decomposition gases has become an urgent need for the assessment of the working state of power equipment, environmental protection, and human health.^14,15^ Over the past few decades, traditional metal oxides have primarily been utilized as sensitive materials for detecting SF_6_ decomposition gases.^16,17^ Nevertheless, these metal oxide sensors exhibit disadvantages, such as a high operating temperature and relatively low sensitivity. Hence, it is essential to design a novel and high-performance gas sensor to monitor or eliminate SF_6_ decomposition gases. In recent years, two-dimensional (2D) nanomaterials have become the primary focus of research in gas-sensing materials due to their unique electronic properties.^18,19^ Moreover, following the great success of graphene as a gas-sensitive material,^20^ the research of various 2D materials such as metal phthalocyanines (MPc),^21–23^ phosphorene,^24,25^ tin disulfide (SnS_2_),^26,27^ and boron nitride (BN)^28^ also shows great research potential.

Recently, porphyrin molecules have been found to exhibit a broad range of physicochemical and biological properties, which render them highly promising for a diverse array of applications in sensors, optoelectronics, and biomedicine.^29–31^ The presence of nitrogen (N) atoms at the center of porphyrins allows them to coordinate with a variety of metal ions and form metalloporphyrin (MPr) complexes. The MPr is capable of combining with gases through hydrogen bonding, van der Waals forces, or interactions with a variety of metal ions.^32,33^ When MPr interacts with gas molecules, their optical and electrical properties undergo significant changes. Therefore, sensors decorated with different MPr materials are considered to be excellent gas sensors.^34–36^ However, two-dimensional (2D) porphyrins doped with a single metal atom still present several challenges, including low selectivity and lack of sensitivity. According to relevant reports, the nitrogen (N) position within the M-N_4_ structure of the catalysts can be substituted by other elements such as boron (B), phosphorus (P), and sulfur (S); this nonmetallic doping can enhance the activity and selectivity of catalysts.^37–39^ Thus, a critical inquiry emerges from this study: whether the incorporation of nonmetallic atoms into two-dimensional metalloporphyrin monolayers can confer remarkable gas-sensing capabilities specifically tailored for SF_6_ decomposition gases.

To address the aforementioned challenges, we commenced our investigation by employing the DFT method to meticulously examine the adsorption and gas-sensing characteristics of SF_6_ decomposition gases on 3d transition metal (M = Sc–Zn) decorated porphyrin monolayers, thereby showcasing our pioneering strategy to augment sensing capabilities. Our initial findings indicated that the NiPr monolayer was ill-suited for sensing SF_6_ decomposition gases, which is attributable to its low adsorption energy and inadequate sensitivity. Building on these insights, we strategically introduced nonmetallic atom dopants, specifically B and O, to modulate the sensing performance of the NiPr monolayer. Consequently, we conducted an exhaustive exploration of the adsorption behavior and gas-sensitive response characteristics of the SF_6_ decomposition gases with the NiBPr and NiOPr monolayers, uncovering significant enhancements in their sensing attributes. The total electron density distribution (TEDD), charge density difference (CDD), and density of states (DOS) were thoroughly analyzed to reveal the microscopic interaction mechanism between the NiBPr and NiOPr monolayers and these gases. Finally, to further establish the applicability of NiBPr and NiOPr monolayers for SF_6_ decomposition gases, the bandgap (*E*_g_), work function (φ), and recovery time (τ) were comprehensively analyzed. This study delivers profound insight into the structural stability and sensitivity of NiBPr and NiOPr monolayers, thereby validating their potential as effective sensors for detecting SF_6_ decomposition gases, underscoring a significant advancement in the field of gas sensing technology.

## 2 Computational Methods and Models

All theoretical calculations were carried out using DFT in the Dmol^3^ program.^40,41^ We utilized the Perdew–Burke–Ernzerhof (PBE) of generalized gradient approximation to deal with the electronic exchange-correlation effects.^42^ The DFT-D approach (Tkatchenko–Scheffler) was utilized to rectify the weak long-range interactions,^43^ which is based on the D2 framework with density-dependent damping. We utilized a double numerical plus polarization (DNP)^44^ atomic orbital basis set and density functional semicore pseudopotentials (DSPP)^45^ for our simulation. To prevent interlayer interactions, a vacuum layer of 20 Å was used.^46,47^ The k-point grids for structure optimization and electronic properties calculation were set to 5 × 5 × 1 and 10 × 10 × 1, respectively.^48,49^ The energy convergence criteria, maximum displacement, and maximum force were 10^–5^ Ha, 5 × 10^–3^ Å, and 2 × 10^–3^ Ha/Å, respectively.^50–52^

In this thesis, to estimate the structural stability of the NiNPr (N = B, O) monolayers, the binding energy (*E*_bin_) was calculated by the following equation:^53–55^

1 $$E_{\mathrm{bin}}=E_{\mathrm{total}}-E_{\mathrm{monolayer}}-E_{m}$$

where *E*_total_ and *E*_monolayer_ are the total energies of the decorated and pure monolayers, and *E*_m_ is the energy of a single Ni atom.

To study the sensitivity of the NiPr and NiNPr (N = B, O) monolayers toward the gases, the adsorption energy (*E*_ads_) of gases on the substrate material is calculated by the following equation:^56,57^

2 $$E_{\mathrm{ads}}=E_{\mathrm{gas}+\mathrm{monolayer}}-E_{\mathrm{monolayer}}-E_{\mathrm{gas}}$$

where *E*_gas+monolayer_ and *E*_monolayer_ are the total energies of the NiPr and NiNPr (N = B, O) monolayers with and without gas adsorption, respectively. *E*_gas_ is the energy of the individual gases. Generally speaking, if *E*_ads_ < 0, it indicates that the adsorption process is exothermic and can occur spontaneously.^58^ In addition to the further description of adsorption ability, the charge transfer (*Q*_t_) between NiPr and NiNPr (N = B, O) monolayers and target gases can be acquired through Hirshfeld charge analysis, in which the *Q*_t_ is defined as^59^

3 $$Q_{t}=Q_{\mathrm{adsorbed‐molecule}}-Q_{\mathrm{isolated‐molecule}}$$

where the *Q*_adsorbed‑molecule_ and *Q*_isolated‑molecule_ are the charges of gases after and before adsorption, respectively. When *Q*_t_ is less than 0, the gases act as electron acceptors and obtain electrons from the monolayer. When *Q*_t_ is greater than 0, the gases act as electron donors, and the electrons are transferred to the monolayer.

## 3 Results and Discussion

### 3.1 Adsorption of SF_6_ Decomposition Gases on Intrinsic NiPr Monolayer

Figure 1 shows that the NiPr monolayer is a two-dimensional metal–organic complex consisting of a central nickel atom (Ni) and a porphyrin ring (Pr). The lattice constant of the NiPr monolayer calculated in this paper is approximately 10.85 Å, which agrees well with previously reported calculation results.^60^ The coplanarity of all atoms in the NiPr monolayer indicates that the NiPr monolayer is completely delocalized and conjugated, as shown in Figure 1a. The bandgap (*E*_g_) of the NiPr monolayer is 0.985 eV in Figure 1b. As displayed in Figure 1c, the density of states (DOS) for the spin-up and spin-down of NiPr is symmetric, indicating that it is a diamagnetic material. Furthermore, there is strong orbital hybridization and a certain number of resonance peaks with the Ni-d orbital and N4-p orbitals within the energy range of −6.25 to 3.75 eV, resulting in a strong binding force between the Ni atom and N atoms. Specifically, the moderate direct bandgap (0.985 eV) of NiPr falls within the optimal range for room-temperature chemiresistive sensing, where charge carrier density is sufficiently tunable upon gas adsorption while maintaining thermal stability. The Ni-centered porphyrin structure provides open coordination sites for gas molecule binding, while the delocalized π-conjugated framework ensures efficient charge transfer between adsorbed species and the sensing layer. These combined characteristics—structural robustness, tunable electronic response, and accessible active sites—establish the fundamental prerequisites for selective gas detection. In conclusion, while the stable configurations and excellent electronic properties of NiPr monolayer suggest theoretical potential, the subsequent adsorption analyses (Section 3.1) reveal weak binding energies (0.12–0.27 eV) and negligible charge transfer, indicating that the pristine NiPr is unsuitable for practical gas sensing applications. This motivates the need for nonmetallic atom modulation strategies discussed in the following sections.

**Figure 1:**
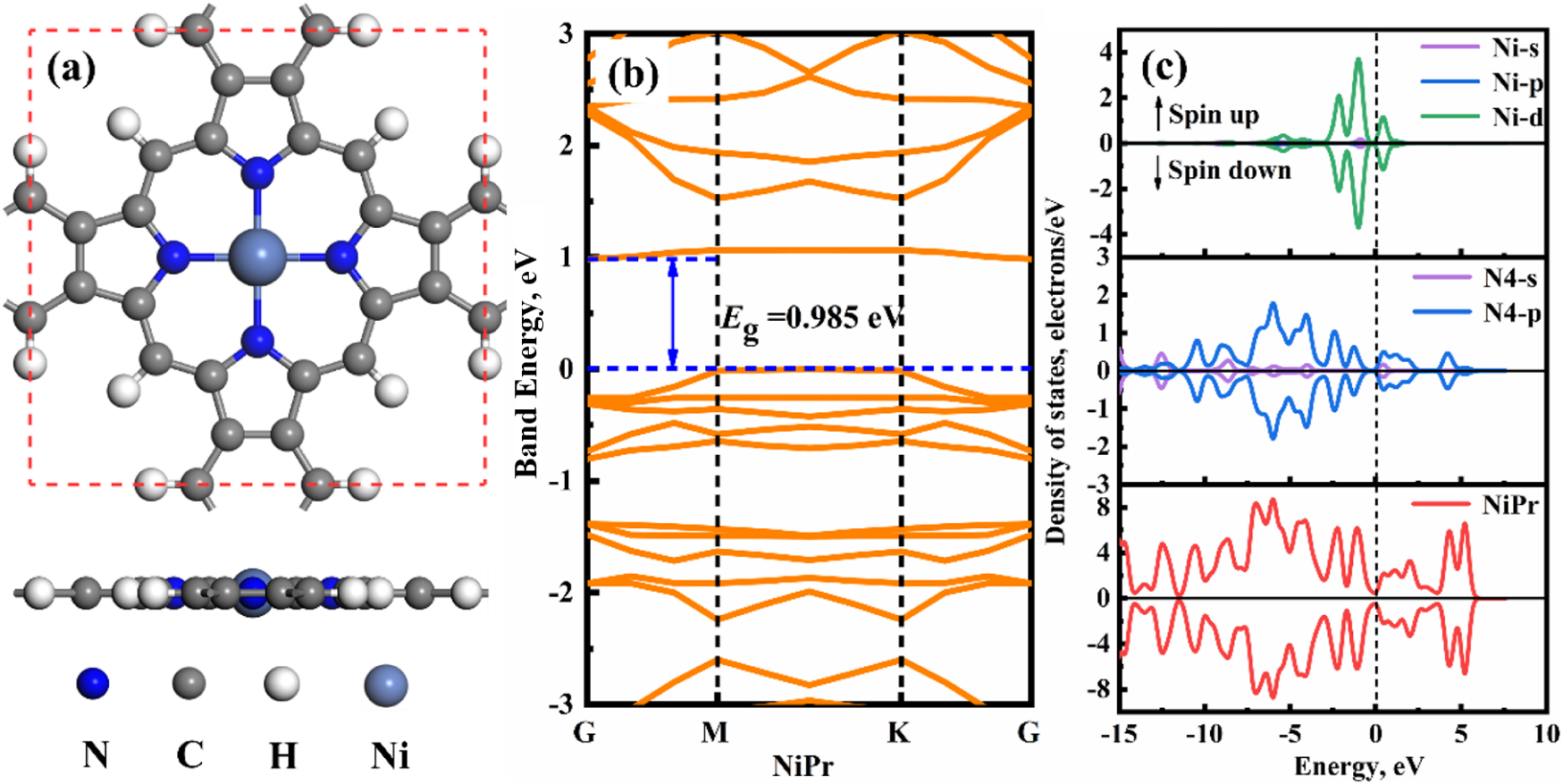
(a) Stable structure, (b) band energy, and (c) DOS of the NiPr monolayer.

The optimized models of gases are depicted in Figure 2. Figure 2a shows that the S–H bond length in H_2_S is 1.357 Å and the H–S–H bond angle is 91.353°, showing a highly symmetrical V-shaped structure. Similar to H_2_S, the SO_2_ gas also exhibits a symmetrical “V”-shaped structure, with the O–S bond length of 1.482 Å and the O–S–O bond angle of 119.879°, as listed in Figure 2b. As shown in Figure 2c, the H–F bond length in the HF gas is 1.090 Å. As depicted in Figure 2d, the F–S–O bond angle in SOF_2_ gas is 107.168°, whereas the F–S–F bond angle is 93.317°. Additionally, the F–S and S–O bond lengths are 1.673 and 1.462 Å, respectively. As illustrated in Figure 2e, the spatial configuration of SO_2_F_2_ gas is a tetrahedral structure. Specifically, the S–O bond length is 1.443 Å, the F–S bond length is 1.615 Å, the F–S–O bond angle is 107.810°, the O–S–O bond angle is 126.431°, and the F–S–F bond angle is 94.383°. Notably, the SO_2_F_2_ gas is symmetric in the plane where the middle vertical lines of two F atoms or two O atoms are connected. In addition, the S–O bond length in SO_2_ (1.482 Å), SOF_2_ (1.462 Å), and SO_2_F_2_ (1.443 Å) is shortened successively, which can be attributed to the gradually enhanced electronegativity of the S atom in the system. In conclusion, the basic parameters of SF_6_ decomposition characteristic gases constructed in this paper are basically consistent with other studies.^61–63^

**Figure 2:**
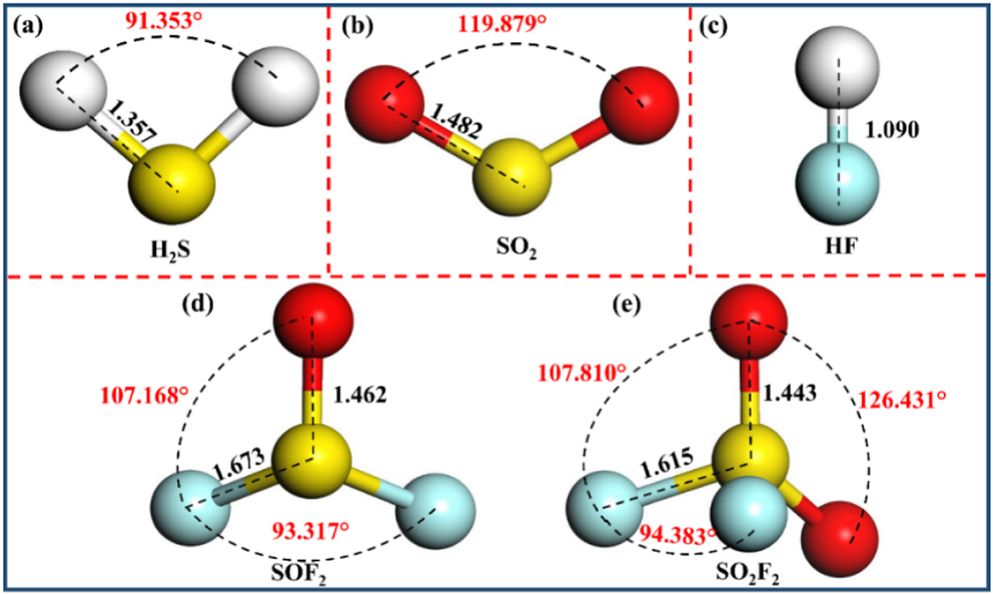
Optimized gas molecules: (a) H_2_S, (b) SO_2_, (c) HF, (d) SOF_2_, and (e) SO_2_F_2_.

To identify the stable adsorption structures of NiPr toward H_2_S, SO_2_, HF, SOF_2_, and SO_2_F_2_ gases, the five types of gases were positioned above the NiPr in different directions. For instance, in the H_2_S, HF, and SO_2_ systems, the gases can be adsorbed on the NiPr surface through the S, F, O, or H atoms in parallel or vertically. Similarly, the SOF_2_ and SO_2_F_2_ gases are adsorbed on the NiPr surface through the S, F, or O atoms, respectively. This adsorption style was also used for NiBPr and NiOPr in this study. Figure 3 displays the most stable adsorption structures in different adsorption systems, while Table 1 lists the adsorption parameters of different adsorption systems. The structures of the NiPr-SO_2_ and NiPr-SOF_2_ are the most stable when the O atom in SO_2_/SOF_2_ is vertically close to the center of the NiPr, as shown in Figure 3a,b. In Figure 3c, the S atom in the H_2_S gas has the best binding effect with the center of the NiPr monolayer. In Figure 3d and e, the HF and SO_2_F_2_ gases are adsorbed on the NiPr monolayer through F atoms. As shown in Table 1, we calculated the adsorption energy (*E*_ads_), charge transfer (Q_t_), adsorption height (*H*_ads_), and bandgap (*E*_g_′) of gas molecules adsorbed on NiPr surfaces. The *E*_g_ refers to the bandgap of the pristine NiPr nanosheet. We found that the interactions between the pristine NiPr monolayer and the gases of SO_2_, H_2_S, SO_2_F_2_, SOF_2_, and HF are considerably weak with *E*_ads_ at −0.12 to −0.27 eV. This suggests that the NiPr monolayer has limited capturing ability for the SO_2_, H_2_S, SO_2_F_2_, SOF_2_, and HF gases. Meanwhile, the SO_2_ gains approximately 0.086 e from the NiPr, while the H_2_S, HF, SOF_2_, and SO_2_F_2_ gases lose approximately 0.021, 0.016, 0.018, and 0.003 e, respectively. Furthermore, the adsorption heights (*H*_ads_) between the NiPr and SO_2_, H_2_S, HF, SOF_2_, and SO_2_F_2_ gases are 3.005, 3.334, 3.052, 3.051, and 2.944 Å, respectively. Notably, there are no significant changes in the bandgap of the NiPr after the adsorption of SO_2_, H_2_S, HF, SOF_2_, and SO_2_F_2_ gases, suggesting that the NiPr monolayer has poor sensitivity toward these gases. Consequently, it can be concluded that the NiPr monolayer is unsuitable as a gas-sensitive material for SO_2_, H_2_S, HF, SOF_2_, and SO_2_F_2_ gases.

**Figure 3:**
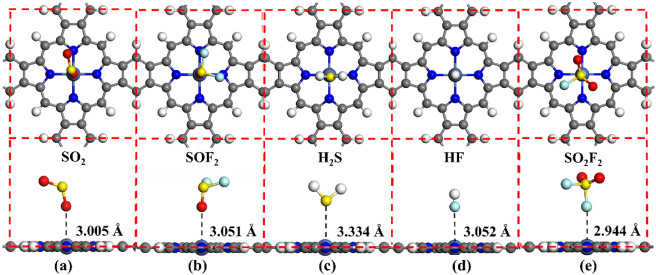
Stable adsorption structures in different adsorption systems: (a) NiPr-SO_2_, (b) NiPr-SOF_2_, (c) NiPr-H_2_S, (d) NiPr-HF, and (e) NiPr-SO_2_F_2_.

**Table 1:** Adsorption Parameters of the SO_2_, H_2_S, HF, SOF_2_, and SO_2_F_2_ Gases on the NiPr Monolayer

Substrate	Gases	*E*_ads_/eV	*Q*_t_/e	*H*_ads_/Å	Donor/Acceptor	*E*_g_′(*E*_g_)/eV
NiPr	SO_2_	–0.27	–0.086	3.005	Acceptor	0.939 (0.985)
	H_2_S	–0.15	0.021	3.334	Donor	0.992 (0.985)
	HF	–0.27	0.016	3.052	Donor	1.000 (0.985)
	SOF_2_	–0.12	0.018	3.051	Donor	0.995 (0.985)
	SO_2_F_2_	–0.18	0.003	2.944	Donor	0.976 (0.985)

Figure 4 depicts the TEDD and CDD for the stable adsorption structures. As illustrated in Figure 4a–e, the TEDD analysis reveals the presence of blank regions between the SO_2_, H_2_S, HF, SOF_2_, and SO_2_F_2_ gases and the NiPr. Consequently, the interaction between these gases and the NiPr monolayer is relatively weak, resulting in poor gas-capturing ability. Furthermore, the CDD analysis reveals that only a minimal amount of electron transfer occurs between the SO_2_, H_2_S, HF, SOF_2_, and SO_2_F_2_ gases and the NiPr monolayer, as shown in Figure 4f–j. The SO_2_ gains electrons from the NiPr monolayer and acts as an electron acceptor. However, the H_2_S, HF, SOF_2_, and SO_2_F_2_ gases each transfer part of their electrons to the NiPr monolayer, thereby acting as electron donors. Overall, the small *Q*_t_ between the NiPr monolayer and the SO_2_, H_2_S, SOF_2_, HF, and SO_2_F_2_ gases further attests to the poor adsorption capacity of the NiPr toward these gases.

**Figure 4:**
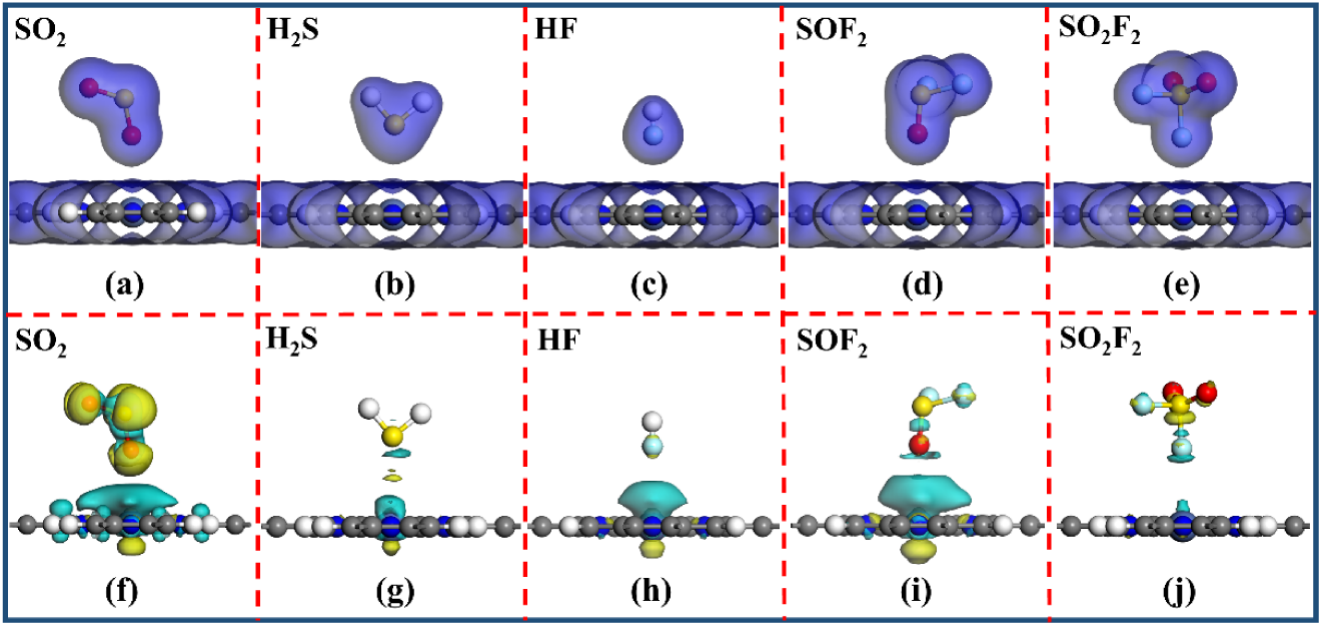
(a–e) TEDD (isovalue: 0.1 e/Å^3^), and (f–j) CDD (isovalue: 0.002 e/Å^3^) of NiPr-SO_2_, NiPr-H_2_S, NiPr-HF, NiPr-SOF_2_, and NiPr-SO_2_F_2_ systems. The yellow (cyan) region is electron accumulation (consumption).

### 3.2 Adsorption of SF_6_ Decomposition Gases on NiBPr Monolayer

The introduction of suitable nonmetallic atomic dopants can significantly improve the chemical activity and sensitivity of nanomaterial gas sensors. Therefore, in this study, we have selected B, O, F, and S atoms, which are widely utilized as nonmetallic atom dopants to modify the chemical properties of the NiPr monolayer, as shown in Figure 5a. In Figure 5b, the binding energy analyses show that the NiBPr and NiOPr monolayers doped with B and O atoms have high binding energy, which subsequently ensures the structural stability of the NiBPr and NiOPr monolayers. The binding energies of the two gas-sensitive materials are in the range of −7.79 to −9.30 eV, and the binding energies are negative, indicating that the substrate structure is thermodynamically stable. Therefore, the subsequent discussions will focus on the NiBPr and NiOPr monolayers.

**Figure 5:**
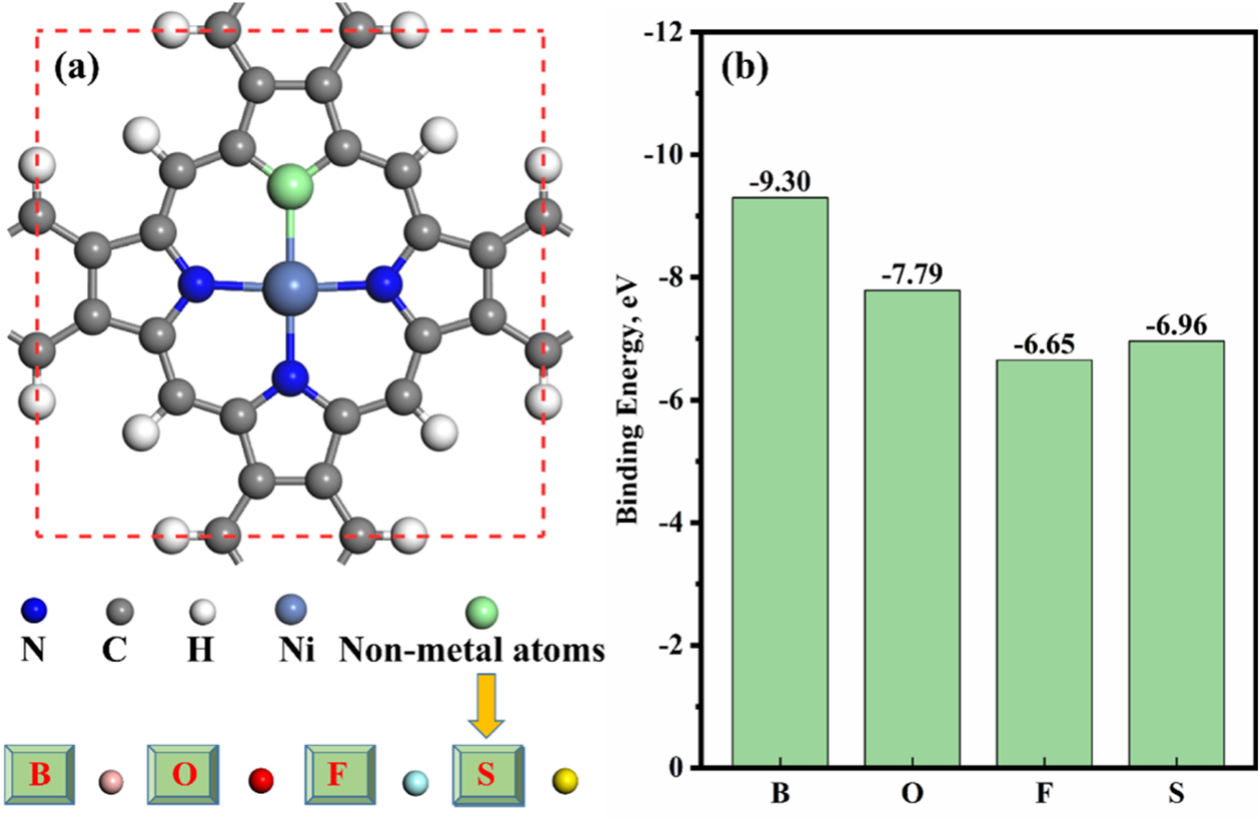
Structures of NiBPr, NiOPr, NiFPr, and NiSPr monolayers (a) and (b) *E*_bin_.

As displayed in Figure 6a–c, the density of states (DOS) for spin-up and spin-down of NiPr/NiBPr/NiOPr is symmetric, indicating that it is a diamagnetic material. Furthermore, there is strong orbital hybridization and a certain number of resonance peaks with the Ni-d orbital and N/B/O-p orbitals within the energy range of −7.15 to 2.75 eV, resulting in a strong binding force between the Ni atom and N/B/O atoms. These indicate that Ni and B/O atoms have a strong bonding interaction.

**Figure 6:**
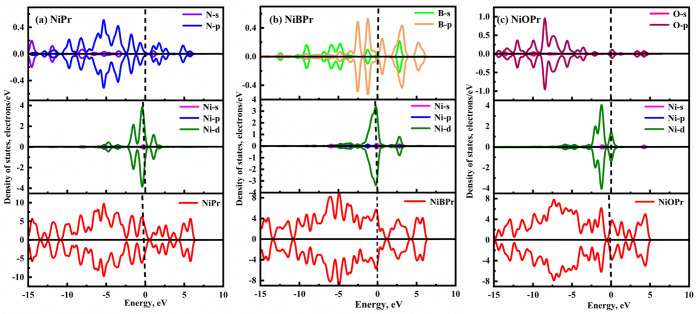
DOS of (a) NiPr, (b) NiBPr, and (c) NiOPr monolayers.

Figure 7 presents the stable adsorption structures for different adsorption systems, while Table 2 lists the adsorption parameters of different adsorption systems. It is observed that H_2_S gas is adsorbed almost parallelly to the NiBPr surface, whereas SO_2_, SO_2_F_2_, and SOF_2_ gases are predominantly vertically adsorbed on the NiBPr monolayer, and HF gas is adsorbed at a certain tilted angle, as illustrated in Figure 7a–e. In Table 2, compared with pure NiPr, the adsorption strength of these five gases on the NiBPr can be improved to different degrees. Among them, the adsorption of SO_2_, H_2_S, HF, and SOF_2_ gases on the NiBPr belongs to chemisorption (*E*_ads_ > 0.5 eV), each exhibiting respective *E*_ads_ values of −0.88 to −0.56 eV, while the adsorption of SO_2_F_2_ gas is physical adsorption and primarily depends on van der Waals forces.^64^ Furthermore, the *H*_ads_ between the NiBPr monolayer and SO_2_, H_2_S, HF, and SOF_2_ gases are 2.282 Å, 2.311 Å, 2.168 Å, and 2.182 Å, respectively. Notably, all of these adsorption heights are smaller than the adsorption height in the NiBPr-SO_2_F_2_ adsorption systems (3.657 Å). Meanwhile, the *Q*_t_ numbers between the NiBPr monolayer and SO_2_, H_2_S, HF, and SOF_2_ gases are −0.171, 0.275, −0.135, and −0.047 e, respectively, and these values are all greater than the *Q*_t_ number (0.034 e) in the NiBPr-SO_2_F_2_ adsorption system. Interestingly, compared to the bandgap of NiPr (0.985 eV), the bandgap is reduced to 0.326 eV after doping with a B atom, which is due to the introduction of bands near the Fermi level region.^65^ Upon the adsorption of SO_2_, H_2_S, HF, and SOF_2_ gases, the bandgap of the NiBPr monolayer undergoes significant changes. However, after the adsorption of SO_2_F_2_ gas, the change in the bandgap of the NiBPr monolayer is negligible, indicating a lower sensitivity to SO_2_F_2_ gas. Therefore, the NiBPr monolayer exhibits suitable adsorption strength and high sensitivity toward SO_2_, H_2_S, HF, and SOF_2_.

**Figure 7:**
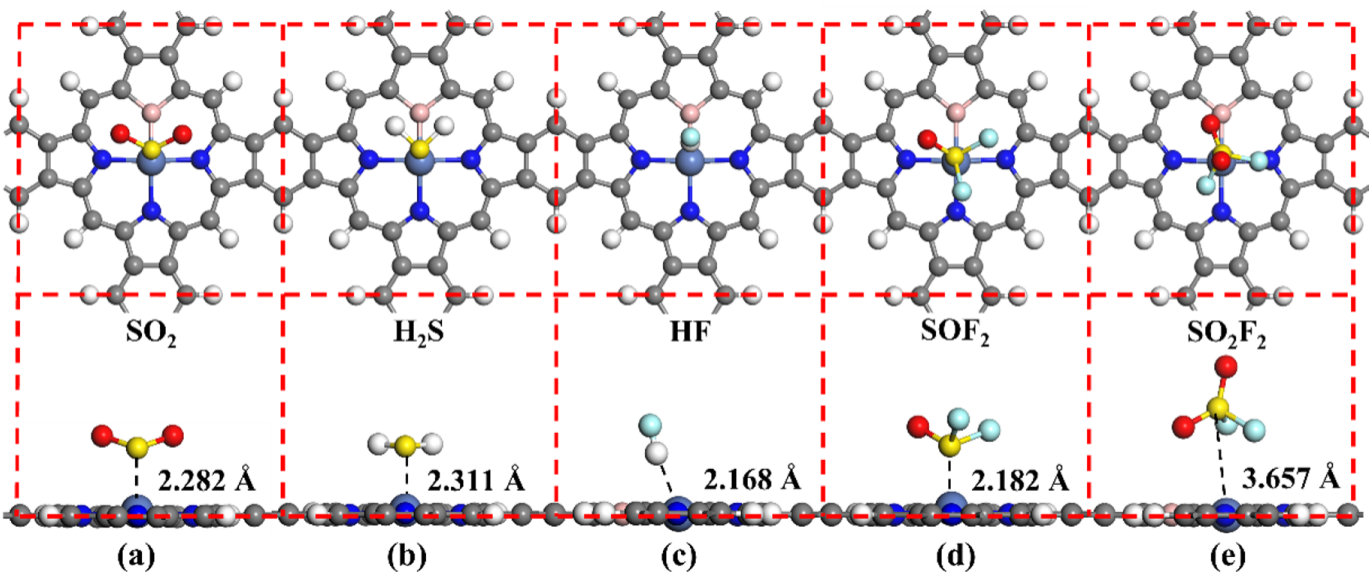
Stable adsorption structures in different adsorption systems: (a) NiBPr-SO_2_, (b) NiBPr-H_2_S, (c) NiBPr-HF, (d) NiBPr-SOF_2_, and (e) NiBPr-SO_2_F_2_.

**Table 2:** Adsorption Parameters of the SO_2_, H_2_S, HF, SOF_2_, and SO_2_F_2_ Gases on the NiBPr Monolayer

Substrate	Gases	*E*_ads_/eV	*Q*_t_/e	*H*_ads_/Å	Donor/Acceptor	*E*_g_′(*E*_g_)/eV
NiBPr	SO_2_	–0.88	–0.171	2.282	Acceptor	0.401 (0.326)
	H_2_S	–0.68	0.275	2.311	Donor	0.556 (0.326)
	HF	–0.56	–0.135	2.168	Acceptor	0.284 (0.326)
	SOF_2_	–0.59	–0.047	2.182	Acceptor	0.539 (0.326)
	SO_2_F_2_	–0.30	0.034	3.657	Donor	0.329 (0.326)

Figure 8 shows the TEDD and CDD of the five gases adsorbed on the NiBPr monolayer. There is a substantial charge overlap between the SO_2_, H_2_S, HF, and SOF_2_ gases and the NiBPr monolayer surface in Figure 8a–d. In contrast, for the NiBPr-SO_2_F_2_ system (Figure 8e), only a minimal number of charges are shared between the SO_2_F_2_ gas and the NiBPr surface. Hence, the NiBPr monolayer exhibits a stronger interaction with the SO_2_, H_2_S, HF, and SOF_2_ gases. The CDD plots reveal that there are obvious electron depletion regions around the NiBPr monolayer (Figure 8f,h,i). In contrast, substantial electron accumulation regions are observed around the SO_2_, HF, and SOF_2_ gases. Similarly, it can be seen from Figure 8g that a large number of electron depletion regions are distributed around the H_2_S gas. Conversely, a large number of electron accumulation regions are formed around the NiBPr monolayer. However, in Figure 8j, there is less *Q*_t_ between the SO_2_F_2_ gas and the NiBPr monolayer, suggesting a weaker interaction between the SO_2_F_2_ gas and the NiBPr monolayer. These results also agree well with the analysis of Hirshfeld charge transfer.

**Figure 8:**
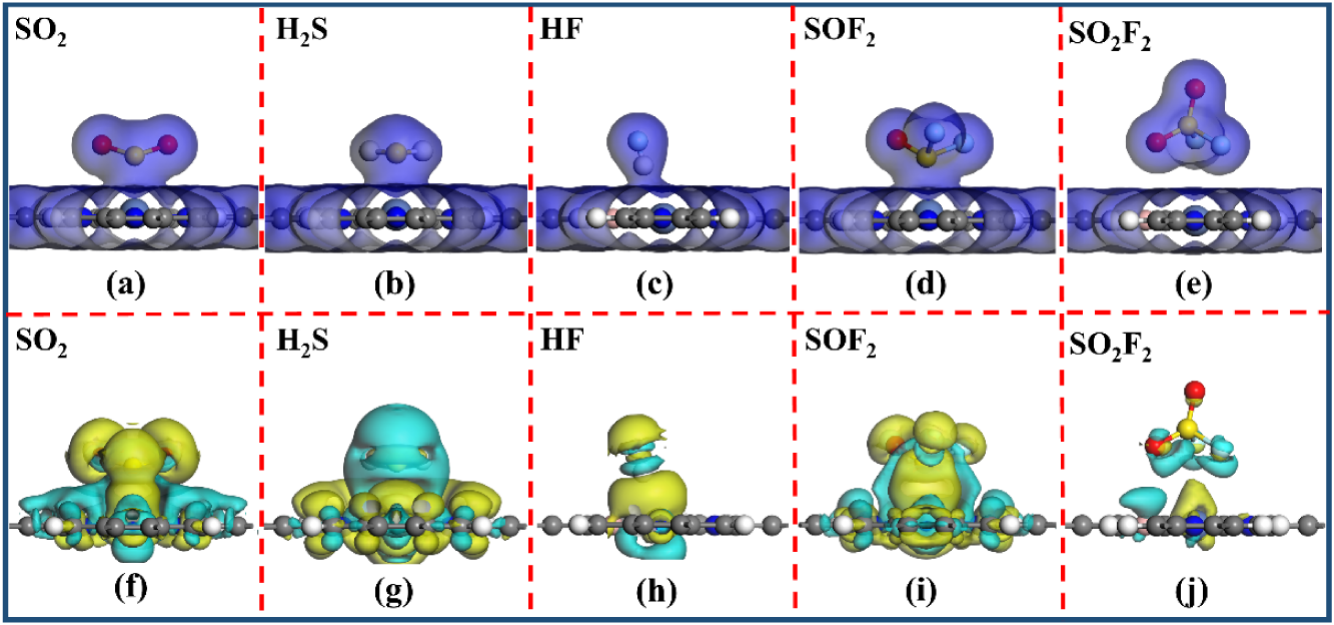
(a–e) TEDD (isovalue: 0.1 e/Å^3^) and (f–j) CDD (isovalue: 0.002 e/Å^3^) of NiBPr-SO_2_, NiBPr-H_2_S, NiBPr-HF, NiBPr-SOF_2_, and NiBPr-SO_2_F_2_ systems. The yellow (cyan) region is electron accumulation (consumption).

Figure 9 depicts the DOS of five various adsorption systems. Figure 9a illustrates that the interaction between SO_2_ and NiBPr is mainly due to the obvious hybridization of the Ni-d orbital and S-p orbitals occurring between −6.85 and 3.55 eV. Additionally, resonance peaks are observed at about −5.19, 0.26, 2.40, 1.33, and 3.05 eV, which further confirm the strong interactions between the NiBPr monolayer and SO_2_ gas. There are several substantial overlap peaks between the Ni-d orbital and S-p orbitals and between the H_2_S-p orbitals and NiN_3_B-d orbitals in the range of −5.44 to 8.75 eV in the NiBPr-H_2_S adsorption system (Figure 9b). This results in the NiBPr monolayer having a strong adsorption strength for H_2_S gas. As can be seen from Figure 9c, there are several significant resonance peaks between the HF gas and the NiBPr monolayer. These peaks are mainly due to the strong hybridizations between the H-s/p orbital and Ni-d orbital at approximately −0.72, 0.38, and 2.38 eV. Similarly, in the NiBPr-SOF_2_ adsorption system (Figure 9d), there are also significant orbital hybridizations between S-s/p and Ni-d. Seven distinct substantial overlap peaks can be observed at −3.97 eV, −0.59, 0.33, 1.47, 2.53, 3.42, and 4.75 eV. Nevertheless, in the NiBPr-SO_2_F_2_ system (Figure 9e), there is no significant hybridization between the S-p/SO_2_F_2_-p orbits and the Ni-d/NiN_3_B-d orbits. This indicates that the orbital hybridization between the NiBPr and SO_2_F_2_ is relatively weak. Compared with the NiBPr-SO_2_F_2_ system, the orbital hybridization of the other four adsorption systems occurs at the Fermi level. Consequently, the adsorption strength of these four adsorption systems is greater.

**Figure 9:**
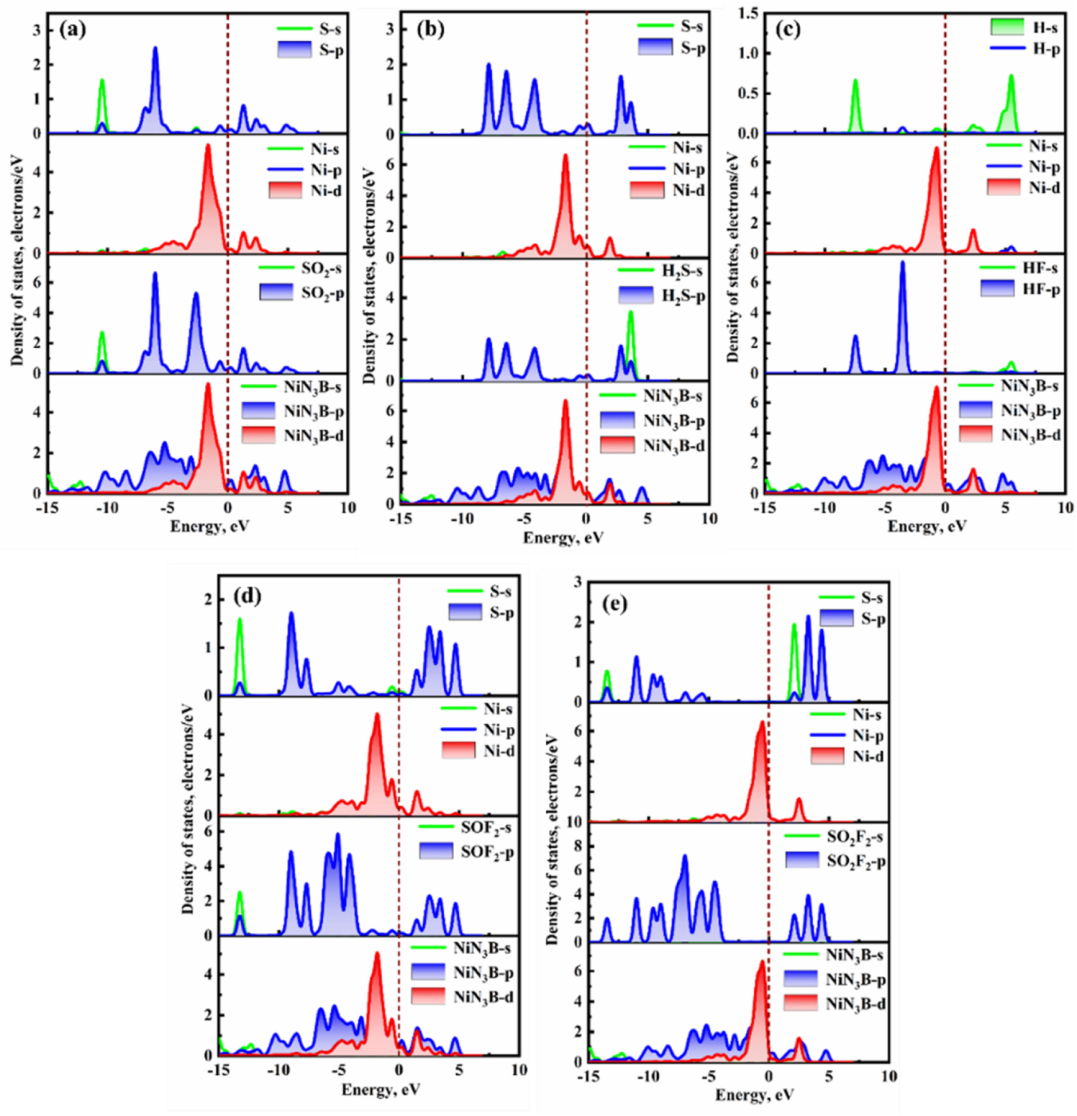
DOS of five different adsorption systems: (a) NiBPr-SO_2_, (b) NiBPr-H_2_S, (c) NiBPr-HF, (d) NiBPr-SOF_2_, and (e) NiBPr-SO_2_F_2_.

### 3.3 Adsorption of SF_6_ Decomposition Gases on NiOPr Monolayer

Figure 10 and Table 3 show the stable adsorption structures and the parameters of five different adsorption systems. From Figure 10a–e, it can be seen that H_2_S is adsorbed nearly parallelly to the NiOPr surface, while SO_2_, HF, and SOF_2_ gases are adsorbed almost perpendicularly to the NiOPr surface, and the SO_2_F_2_ gas is adsorbed at a certain tilted angle. As can be seen from Table 3, the NiOPr monolayer results in varying degrees of enhancement in its adsorption strength toward SO_2_, H_2_S, SOF_2_, HF, and SO_2_F_2_ gases. Similarly, the adsorption of SO_2_, H_2_S, HF, and SOF_2_ by the NiOPr belongs to chemisorption (*E*_ads_ > 0.5 eV), and the corresponding *E*_ads_ are −1.01, −0.58, −0.67, and −0.73 eV, respectively. It is important to note that while our calculations assume molecular adsorption, the strong chemisorption of SO_2_ on NiOPr (−1.01 eV) suggests a risk of dissociative adsorption or surface poisoning under operational conditions, potentially requiring thermal regeneration. These *E*_ads_ were significantly greater than the NiOPr-SO_2_F_2_ system (−0.45 eV). Moreover, the *H*_ads_ of SO_2_, H_2_S, HF, and SOF_2_ gases on the NiOPr monolayer are 2.400 Å, 2.555 Å, 2.105 Å, and 2.537 Å, respectively, and the corresponding *Q*_t_ values are −0.243, 0.157, −0.135, and −0.143 e, respectively. Thus, the NiOPr monolayer has a strong affinity for SO_2_, H_2_S, HF, and SOF_2_ gases. Interestingly, the *E*_g_ of NiOPr after doping with O atoms decreases to 0.057 eV compared to the *E*_g_ of NiPr (0.985 eV), which is due to the introduction of bands near the Fermi level region.^65^ With the adsorption of H_2_S, SO_2_, HF, and SOF_2_ gases, the *E*_g_ of the NiOPr changes to different degrees. To sum up, the NiOPr monolayer possesses both suitable adsorption strength and high sensitivity to SO_2_, H_2_S, HF, and SOF_2_ gases.

**Figure 10:**
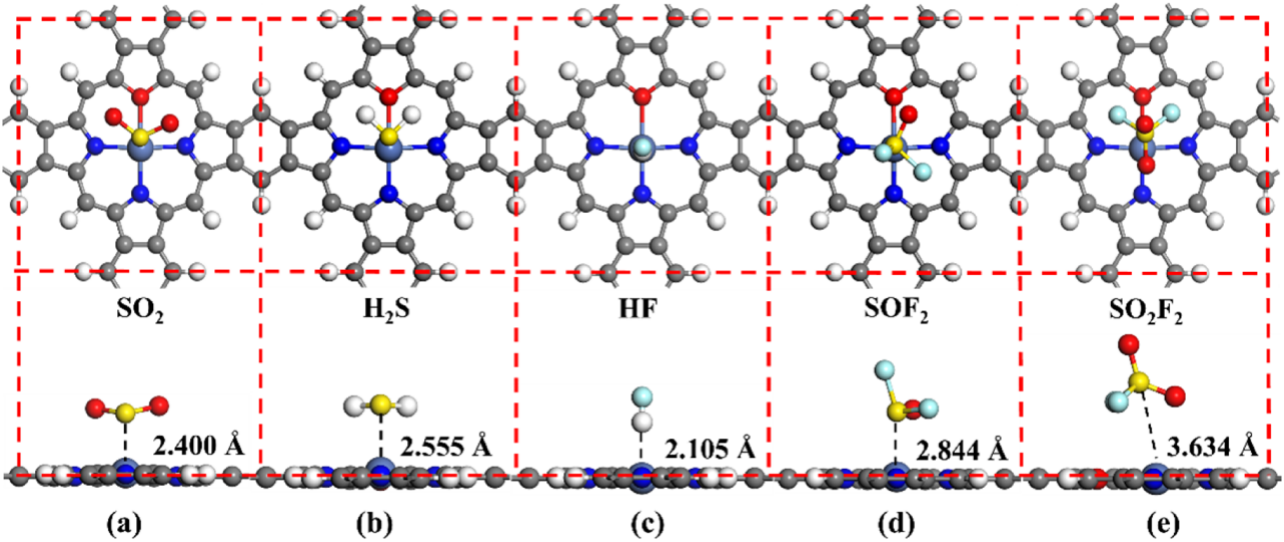
Stable adsorption structures in different adsorption systems: (a) NiOPr-SO_2_, (b) NiOPr-H_2_S, (c) NiOPr-HF, (d) NiOPr-SOF_2_, and (e) NiOPr-SO_2_F_2_.

**Table 3:** Adsorption Parameters of the SO_2_, H_2_S, HF, SOF_2_, and SO_2_F_2_ Gases on the NiOPr Monolayer

Substrate	Gases	*E*_ads_/eV	*Q*_t_/e	*H*_ads_/Å	Donor/Acceptor	*E*_g_′(*E*_g_)/eV
NiOPr	SO_2_	–1.01	–0.250	2.400	Acceptor	0.921 (0.057)
	H_2_S	–0.58	0.157	2.555	Donor	0.028 (0.057)
	HF	–0.67	–0.135	2.105	Acceptor	0.640 (0.057)
	SOF_2_	–0.73	–0.143	2.537	Acceptor	0.760 (0.057)
	SO_2_F_2_	–0.45	0.027	3.634	Donor	0.050 (0.057)

Figure 11 shows the corresponding TEDD and CDD diagrams for five different adsorption systems. It can be observed from Figure 11a–d that there is a significant charge overlap distribution between the SO_2_, H_2_S, HF, and SOF_2_ gases and the NiOPr monolayer. In contrast, there is little charge overlap between the SO_2_F_2_ gas and the NiOPr monolayer (Figure 11e). Consequently, the NiOPr monolayer exhibits a stronger interaction with the SO_2_, H_2_S, HF, and SOF_2_ gases. As shown in Figure 11f,h,i, the CDD plots reveal that there are obvious electron depletion regions around the NiOPr monolayer. In contrast, substantial electron accumulation regions are observed around the SO_2_, HF, and SOF_2_ gases. Similarly, it can be seen from Figure 11g that a large number of electron depletion regions are distributed around the H_2_S gas. Conversely, a large number of electron accumulation regions exist around the NiOPr monolayer. In the NiOPr-SO_2_F_2_ adsorption system (Figure 11j), there is less *Q*_t_ between the SO_2_F_2_ gas and the NiOPr monolayer, suggesting that the interaction between SO_2_F_2_ gas and NiOPr is relatively weak. Compared with the NiOPr-SO_2_F_2_ adsorption system, the significant *Q*_t_ further validates the stronger interaction between the four toxic gases of H_2_S, SO_2_, HF, and SOF_2_ and the NiOPr monolayer.

**Figure 11:**
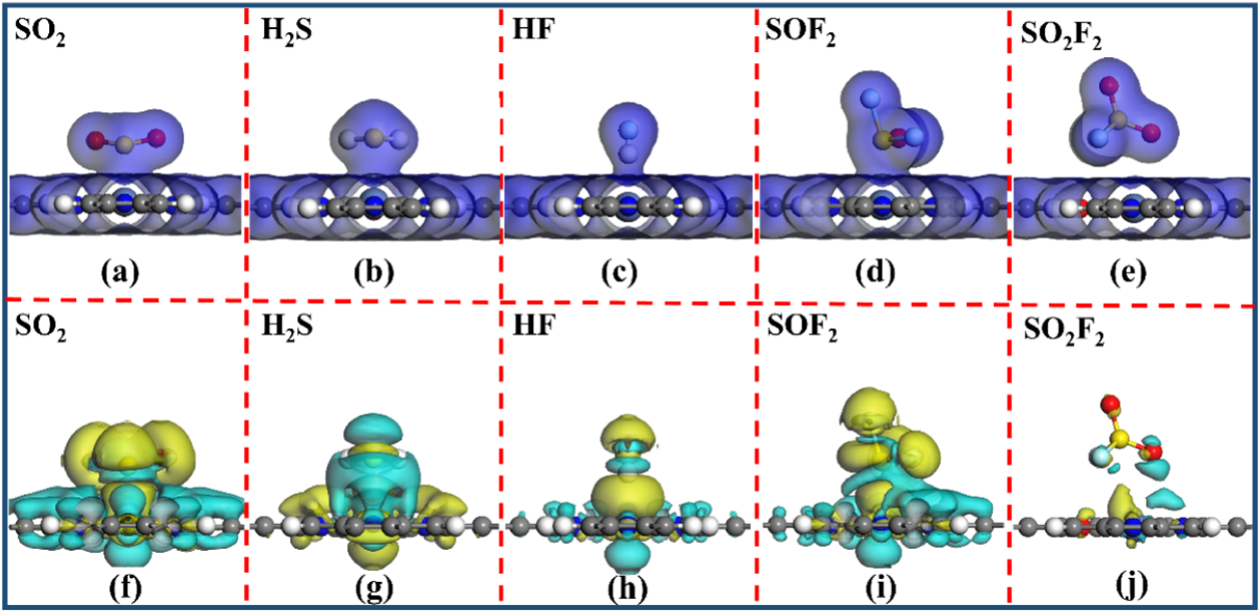
(a–e) TEDD (isovalue: 0.1 e/Å^3^), and (f–j) CDD (isovalue: 0.002 e/Å^3^) of NiOPr-SO_2_, NiOPr-H_2_S, NiOPr-HF, NiOPr-SOF_2_, and NiOPr-SO_2_F_2_ systems. The yellow (cyan) region is the electron accumulation (consumption).

Figure 12 depicts the DOS values of different adsorption systems. In the NiOPr-SO_2_ adsorption system (Figure 12a), there is a significant orbital hybridization between the S-p orbital of SO_2_ and the Ni-d orbital of the NiOPr monolayer in the range of −7.22 to −2.56 eV. Furthermore, there are prominent substantial overlap peaks at approximately −2.31 eV, −0.94 eV, and 1.47 eV. Similarly, the SO_2_-s/p orbitals also undergo significant orbital hybridization with NiN_3_O-d orbitals in the above energy range, resulting in a strong chemical interaction between SO_2_ gas and NiOPr. For the NiOPr-H_2_S adsorption systems (Figure 12b), the S-s/p orbitals and Ni-d orbitals primarily exhibit significant orbital hybridization in the range from −6.44 to 4.75 eV. Additionally, the H_2_S-p orbitals and NiN_3_O-d orbitals exhibit three strong resonance peaks at approximately −3.57 eV, −0.40 eV, and 3.59 eV. Similarly, in the NiOPr-HF and NiOPr-SOF_2_ adsorption systems (Figure 12c,d), there is also significant orbital hybridization occurring between H-s/S-p and Ni-d orbitals in the range of −5.00 to 5.30 eV. However, in the NiOPr-SO_2_F_2_ adsorption system (Figure 12e), the hybridization between S-p and Ni-d orbitals is comparatively weak. Moreover, there is no orbital interaction between the NiN_3_O-p/d orbitals and SO_2_F_2_-s/p at the Fermi level, thus indicating that NiOPr interacts weakly with SO_2_F_2_ gas. In summary, the NiOPr monolayer displays remarkable capturing ability for H_2_S, SO_2_, HF, and SOF_2_ gases.

**Figure 12:**
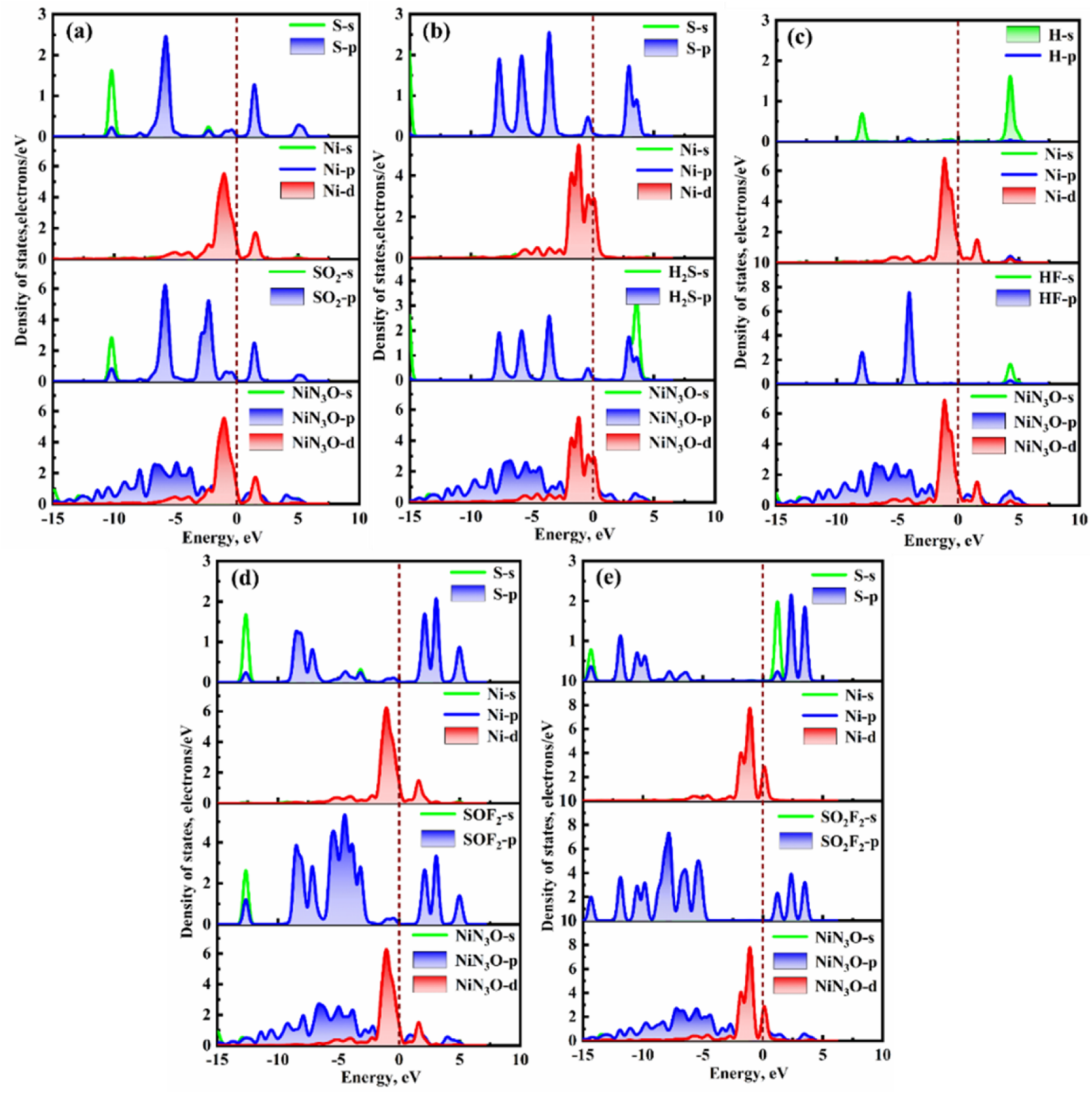
DOS of different adsorption systems: (a) NiOPr-SO_2_, (b) NiOPr-H_2_S, (c) NiOPr-HF, (d) NiOPr-SOF_2_, and (e) NiOPr-SO_2_F_2_.

### 3.4 Selectivity of NiBPr and NiOPr Monolayers

Gas sensors typically operate under atmospheric conditions; hence, the selectivity of NiBPr and NiOPr monolayer films toward the decomposition products of SF_6_ is a critical research focus and an important evaluation criterion. Consequently, it is necessary to investigate the impact of oxygen (O_2_) and water vapor (H_2_O) molecules on the adsorption characteristics of NiBPr and NiOPr monolayers. The adsorption properties of the gases O_2_ and H_2_O on the monolayers of NiBPr and NiOPr are shown in Table 4. It is evident that the adsorption energies of H_2_O and O_2_ on NiBPr and NiOPr monolayers are −0.41 eV to −0.29 eV, respectively, indicating that the NiBPr and NiOPr monolayers have limited capturing ability for the H_2_O and O_2_ molecule. Furthermore, the adsorption heights between NiBPr and NiOPr monolayers and H_2_O and O_2_ molecules are 2.604, 2.042, 3.077, and 4.889 Å. Meanwhile, the *Q*_t_ between NiBPr/NiOPr monolayers and H_2_O and O_2_ molecule are 0.052 e, 0.096 e, 0.008 e, and 0.103 e, respectively, which are all minor than those in the other adsorption systems. This indicates that the NiBPr/NiOPr monolayer exhibits good selectivity for the decomposition products of SF_6_ molecules, and its selectivity remains unaffected in the presence of O_2_ and H_2_O.

**Table 4:** Adsorption Parameters of the H_2_O and O_2_ on the NiBPr/NiOPr Monolayers

Substrate	Gases	*E*_ads_/eV	*Q*_t_/e	*H*/Å	*E*_g_′(*E*_g_)/eV
NiBPr	H_2_O	–0.38	0.052	2.604	0.351 (0.326)
	O_2_	–0.41	–0.096	2.042	0.000 (0.326)
NiOPr	H_2_O	–0.40	0.008	3.077	1.057 (0.057)
	O_2_	–0.29	–0.103	4.889	0.600 (0.057)

### 3.5 Sensing Performance Evaluation

In gas sensors, variation in the electrical conductivity (σ) is a key factor in determining the sensing action. Generally, the change of *E*_g_ is beneficial to evaluate the change of the electrical conductivity (σ) within the adsorption system. As a result, to further infer the sensitivity of NiBPr and NiOPr monolayers to the five gases, σ can be obtained by^56,66^

4 $$\sigma \propto \exp\left(\frac{-E_{g}}{2K_{B}T}\right)$$

where *K*_B_ means the Boltzmann’s constant (8.62 × 10^–5^ eV K^–1^), and *T* means the temperature, respectively.

Apart from σ, the work function (φ) is one of the most important parameters to evaluate the sensitivity of a gas sensor. It can be utilized to assess the sensitivity of a material toward the gases. The value of φ can be obtained by^67,68^

5 $$\varphi =E_{\mathrm{vac}}-E_{\mathrm{Fer}}$$

where *E*_vac_ is the vacuum level, and *E*_Fer_ is the Fermi level, respectively.

The φ values of the five different gases adsorbed on the NiBPr and NiOPr monolayers are shown in Figure 13. The φ values for the clean NiBPr and NiOPr monolayers are 4.83 and 4.58 eV, respectively. Following the adsorptions of SO_2_, H_2_S, HF, and SOF_2_ gases, the φ values for NiBPr change to 5.57, 3.87, 5.57, and 5.33 eV, respectively. However, the adsorption of SO_2_F_2_ gas has little effect on the φ of the NiBPr monolayer, implying that NiBPr is not sensitive to SO_2_F_2_ gas. Consequently, NiBPr exhibits high sensitivity to SO_2_, H_2_S, HF, and SOF_2_ gases. Similarly, following the adsorption of SO_2_, H_2_S, HF, and SOF_2_ gases, the φ of the NiOPr monolayer changes to 5.47, 4.02, 5.02, and 5.40 eV, respectively. The changes in φ due to the adsorption of four gases are much larger than those caused by SO_2_F_2_ gas, which demonstrates that the NiBPr and NiOPr monolayers could potentially be utilized as φ gas-sensitive material for detecting SO_2_, H_2_S, HF, and SOF_2_ gases.

**Figure 13:**
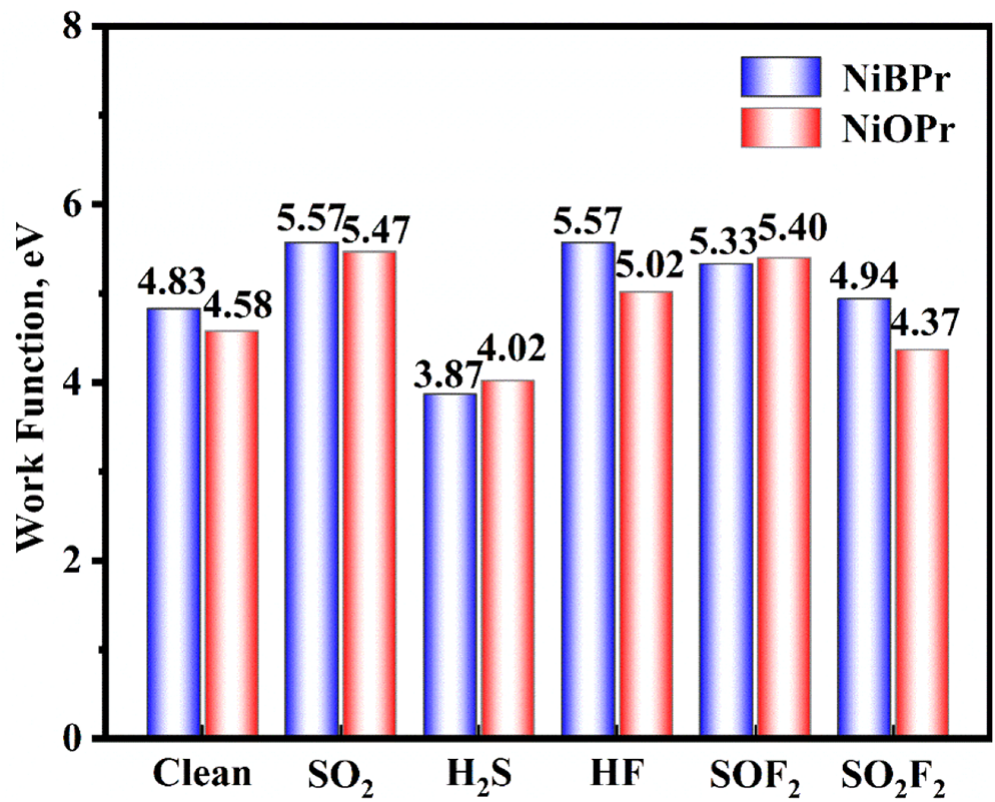
φ of the five different gases adsorbed on the NiBPr and NiOPr monolayers.

To ensure consistent evaluation, we employ dual criteria for sensing assessment: (i) binding strength (|*E*_ads_| > 0.5 eV indicates chemisorption suitable for stable detection) and (ii) electronic perturbation (Δ*E*_g_). Figures 14 and 15 show the band structures of different adsorption systems. One can see that the NiBPr and NiOPr monolayers undergo various changes in their bandgap after the adsorption of five gases. From Figure 14a–d, after the adsorption of SO_2_, H_2_S, HF, and SOF_2_, the NiBPr monolayer exhibits bandgaps of 0.401, 0.556, 0.284, and 0.539 eV, respectively. However, the bandgap change induced by the adsorption of SO_2_F_2_ in the NiBPr monolayer is negligible (0.329 eV). This indicates that the NiBPr material exhibits excellent sensitivity to H_2_S, SO_2_, HF, and SOF_2_. For NiBPr, although HF (Δ*E*_g_ = 0.042 eV) and SO_2_F_2_ (Δ*E*_g_ = 0.003 eV) show similar bandgap changes, their binding energies differ significantly (−0.56 eV vs −0.30 eV), distinguishing chemisorption (reversible sensing) from physisorption (weak response). Similarly, from Figure 15a–e, the bandgaps of NiOPr changed to 0.921, 0.028, 0.640, 0.760, and 0.050 eV for SO_2_, H_2_S, HF, SOF_2_, and SO_2_F_2_ gases, respectively. Due to the low adsorption capacity of NiOPr for SO_2_F_2_ gas, it can be seen that NiOPr is expected to become a gas-sensitive material for the detection of SO_2_, H_2_S, HF, and SOF_2_ gases.

**Figure 14:**
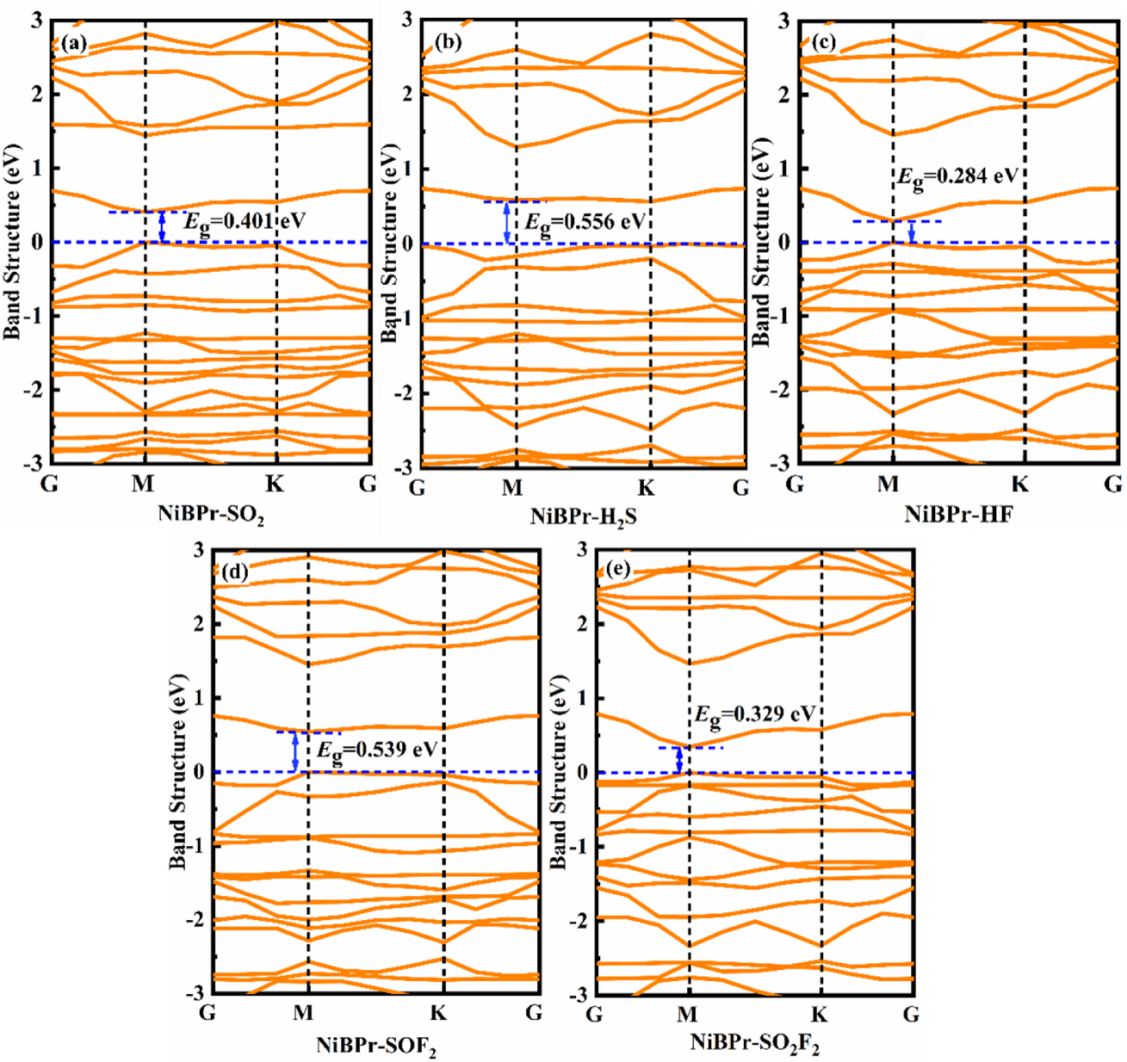
Band structures of different adsorption systems: (a) NiBPr-SO_2_, (b) NiBPr-H_2_S, (c) NiBPr-HF, (d) NiBPr-SOF_2_, and (e) NiBPr-SO_2_F_2_.

**Figure 15:**
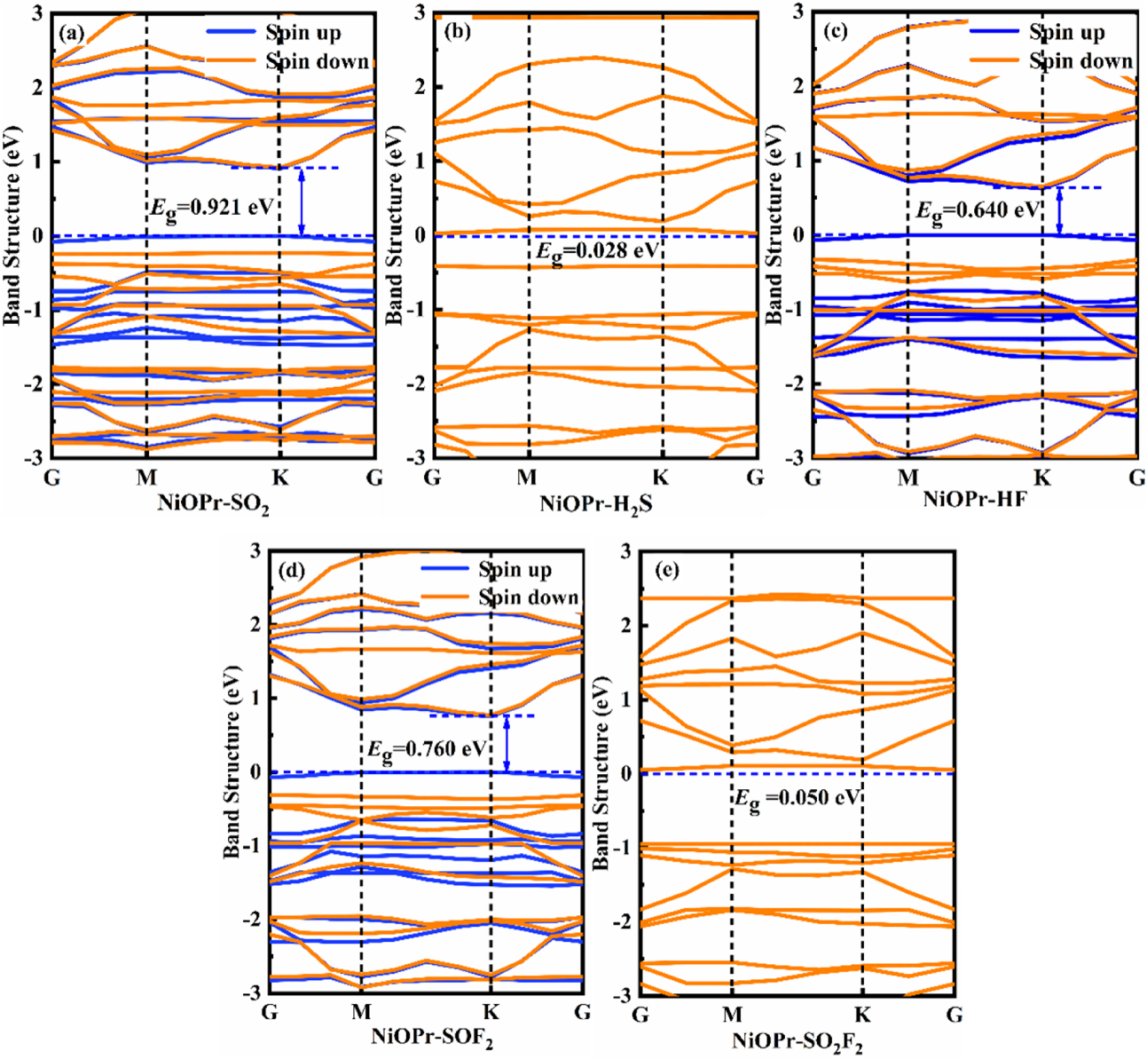
Band structures of different adsorption systems: (a) NiOPr-SO_2_, (b) NiOPr-H_2_S, (c) NiOPr-HF, (d) NiOPr-SOF_2_, and (e) NiOPr-SO_2_F_2_.

Gas adsorption induces magnetization in the otherwise diamagnetic NiOPr monolayer. This magneto-electric response provides a secondary sensing dimension: the material becomes magnetic only in the presence of specific gases (e.g., NiOPr-SO_2_, NiOPr-HF), enabling multimodal detection combining resistive and magnetic signals. The spintronic response offers additional selectivity, as gases with similar binding energies but different spin-coupling characteristics produce distinct magnetic signatures.

During the desorption process, the gas molecules must overcome the energy barrier to be removed. To achieve an optimal desorption performance in gas-sensitive applications, it is crucial to maintain the adsorption energy at a suitable value. Very low adsorption energy hinders gas molecules from adsorbing on the sensing material due to insufficient interaction. Furthermore, excessively strong adsorption energy can hinder gas desorption and sensor reusability. So, to study the recovery time (τ) of NiBPr and NiOPr for these gases, the τ can be calculated as^69^

6 $$\tau =\upsilon_{0}^{-1}\exp\left(\frac{-E_{ads}}{K_{B}T}\right)$$

where *K*_B_ means the Boltzmann’s constant (8.62 × 10^–5^ eV K^–1^), *T* signifies the temperature, and υ_0_ (10^12^ s^–1^) is the attempt frequency. Note that this Arrhenius-type estimation assumes that the desorption barrier equals the adsorption energy (*E*_ads_), which may underestimate τ for strong chemisorption systems where orbital reorganization barriers exist. The reported values, therefore, represent theoretical lower bounds for recovery time.

Figure 16 shows the τ values of five gases by NiBPr and NiOPr at three different operating temperatures. As can be seen from the horizontal comparison of Figure 16a, when the temperature gradually rises, the corresponding τ for each gas adsorption system tends to decrease. However, when we compare the vertical comparison, the τ is SO_2_F_2_ < HF < SOF_2_ < H_2_S < SO_2_. At the normal operating temperature (298–498 K), the τ of SO_2_F_2_ gas by the NiBPr monolayer is remarkably short, indicating that SO_2_F_2_ gas is readily released from the NiBPr monolayer after adsorption. At 298 K, the τ of HF, SOF_2_, H_2_S, and SO_2_ is 2.94 × 10^–3^ s, 9.44 × 10^–3^ s, 3.14 × 10^–1^ s, and 7.55 × 10^2^ s, respectively. Given the favorable adsorption capacity and desorption time of the NiBPr monolayer, it is capable of repeatedly detecting SO_2_, H_2_S, HF, and SOF_2_ gases. Similarly, in Figure 16b, the τ value of all adsorption systems decreases significantly when the temperature reaches 498 K. As illustrated in the red bar graph, the τ of the SO_2_F_2_ gas adsorption system is remarkably short at different temperatures, indicating that the SO_2_F_2_ gas does not undergo chemical adsorption with the NiBPr monolayer. The NiOPr-SO_2_ adsorption system exhibits a long τ of 1.19 × 10^5^ s at 298 K, indicating that SO_2_ gas fails to desorb from the NiOPr monolayer at normal temperature. However, the τ for SO_2_ is 6.10 s at 398 K and 1.65 × 10^–2^ s at 498 K. Consequently, we can conclude that the NiOPr monolayer exhibits excellent sensing and response capabilities toward SO_2_ gas at high temperatures. Compared with the NiOPr-SO_2_F_2_ adsorption systems (298 K), the NiOPr has a short τ at 298 K, up to 6.40 × 10^–3^ s, 2.13 × 10^–1^ s, and 2.20 s for the H_2_S, HF, and SOF_2_, respectively. The short desorption time suggests that the NiOPr has the potential to function as a reusable gas sensing material for H_2_S, HF, and SOF_2_ at room temperature.

**Figure 16:**
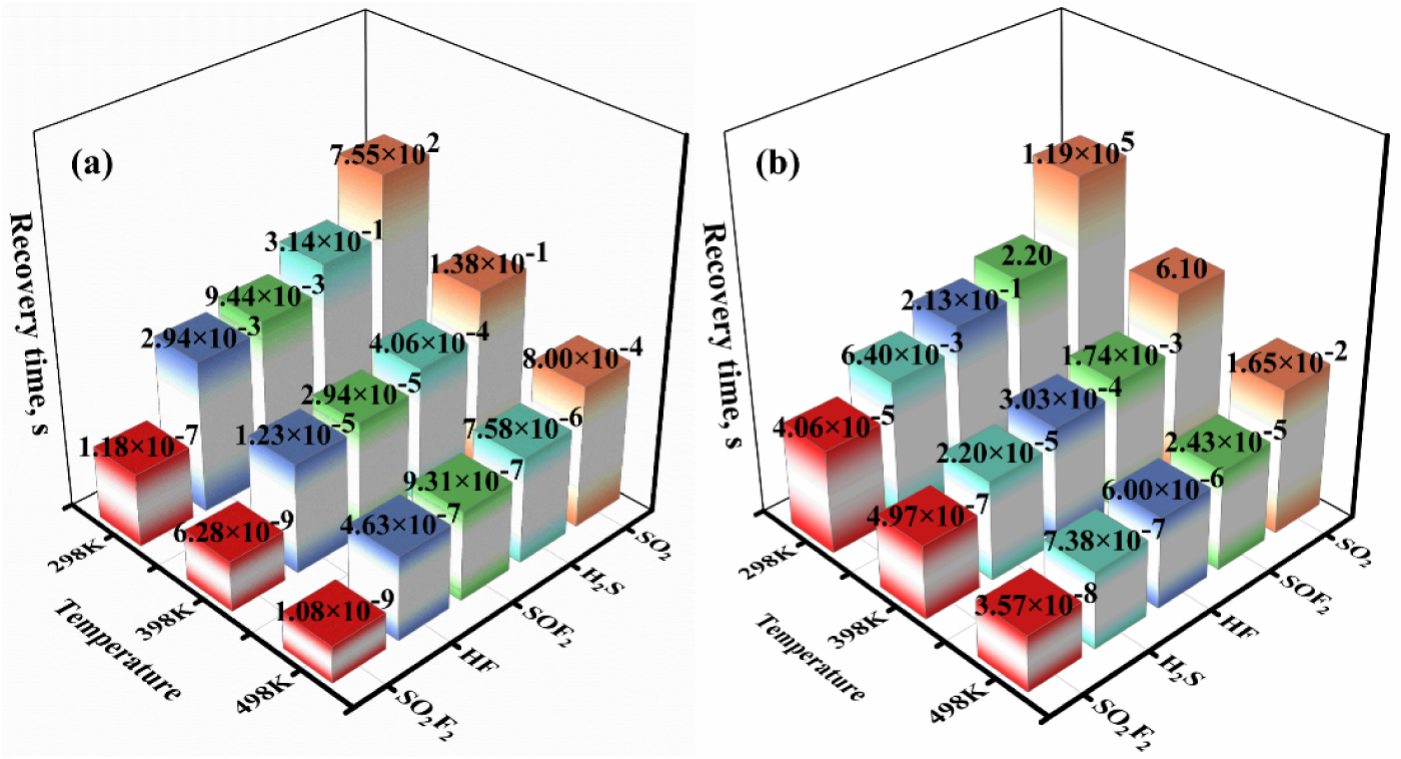
Recovery time of (a) NiBPr and (b) NiOPr monolayers toward the SF_6_ decomposition products at different temperatures.

Finally, to further substantiate that the NiBPr and NiOPr monolayers are promising for detecting SF_6_ decomposed gases, we compared the findings of this study with the adsorption results observed for SF_6_ decomposition gases on other materials. As shown in Table 5, it can be observed that NiBPr and NiOPr monolayers exhibit a significant chemical reactivity toward SO_2_, H_2_S, HF, and SOF_2_ gases compared to most other materials, showing strong *E*_ads_, shorter *H*, and obvious *Q*_t_. By comparing the adsorption parameters of SO_2_F_2_ gas on CoO-SnSe, Tellurene, and Au-SnS_2_ materials, it can be found that NiBPr and NiOPr monolayers have better adsorption characteristics compared with those materials. In conclusion, the NiBPr and NiOPr monolayers have better adsorption properties than other materials, and excellent gas-sensitive response capabilities offer great research potential in the field of chemical gas sensors.

**Table 5:** Comparison of the Adsorption Characteristics of Different Substrates for the SF_6_ Decomposition Gases

Gases	Substrates	*E*_ads_/eV	*Q*_t_/e	*H*/Å	References
SO_2_	NiBPr	–0.88	–0.171	2.282	This work
	NiOPr	–1.01	–0.250	2.400	This work
	Pd-HfSe_2_	–0.11	–0.046	3.730	^42^
	Tellurene	–0.382	–0.158	3.278	^70^
	SbN	–0.50	–0.192	3.140	^71^
H_2_S	NiBPr	–0.68	0.257	2.318	This work
	NiOPr	–0.58	0.157	2.555	This work
	Au-GaNNT	–0.234	0.072	2.720	^2^
	SbN	–0.30	–0.045	2.910	^71^
	Tellurene	–0.317	0.026	1.400	^70^
HF	NiBPr	–0.56	0.135	2.168	This work
	NiOPr	–0.67	–0.135	2.105	This work
	Cu-InN	–0.09	–0.14	2.250	^72^
	Ag-GaN	–0.267	–0.062	2.402	^9^
	Tellurene	–0.342	–0.016	3.919	^70^
	CoO-SnSe	–0.433	–0.024	3.824	^1^
SOF_2_	NiBPr	–0.59	–0.047	2.182	This work
	NiOPr	–0.73	–0.143	2.537	This work
	Au-SnS_2_	–0.254	–0.391	2.645	^73^
	SbN	–0.31	–0.062	3.620	^71^
	Tellurene	–0.352	–0.093	3.590	^70^
SO_2_F_2_	NiBPr	–0.45	0.039	3.657	This work
	NiOPr	–0.30	0.027	3.634	This work
	CoO-SnSe	–0.021	–0.014	2.190	^1^
	Tellurene	–0.231	–0.007	3.798	^70^
	Au-SnS_2_	–0.154	–0.4097	2.671	^73^

## 4 Conclusion

This study presents a systematic investigation into the adsorption characteristics of five distinct gases on NiPr, NiBPr, and NiOPr monolayers through the application of DFT calculations. By dissecting parameters such as *E*_ads_, DOS, CDD, φ, and τ, we unveiled the intrinsic sensing mechanisms of the NiBPr and NiOPr monolayers in response to these gases. While theoretical analysis supports molecular adsorption and reversible sensing for moderate binding cases, strong chemisorption systems may require thermal regeneration or exhibit gradual deactivation through surface poisoning. Experimental validation of cycling stability is essential to confirm reusability. The standout findings of our research are succinctly summarized as follows:

1 The intrinsic NiPr monolayer exhibits low adsorption energy and poor sensitivity, making it unsuitable as a gas-sensitive material for the detection of these five gases.
2 Compared to the bandgap of the pristine NiPr monolayer (0.985 eV), the doping of nonmetallic B and O atoms leads to a reduction in the bandgap of the NiBPr and NiOPr monolayers.
3 After the doping of the nonmetallic B and O atoms, the adsorption strength of H_2_S, SO_2_, HF, and SOF_2_ gases on the NiBPr and NiOPr monolayers can be enhanced to different degrees, and both exhibit chemical adsorption properties toward these four gases.
4 The NiBPr and NiOPr monolayers have significant gas capture capabilities, mainly due to the strong hybridization of the NiN_3_B/NiN_3_O-d orbitals with the gas s/p orbitals.
5 Owing to favorable adsorption/desorption dynamics, the NiBPr monolayer exhibits potential as a recyclable gas-sensitive material for H_2_S, SO_2_, HF, and SOF_2_. Similarly, the NiOPr monolayer demonstrates potential for reusable gas sensing (H_2_S, HF, SOF_2_) and high-temperature SO_2_ monitoring.

## Data Availability

The data that support the findings of this study are available within the article.
